# 576. Determinants of COVID-19 Vaccine Hesitancy: A Cross-Sectional Study in 3 Communities in the United States and Lebanon

**DOI:** 10.1093/ofid/ofab466.774

**Published:** 2021-12-04

**Authors:** Mohamad Yasmin, Mohamad Ali Tfaily, Rebecca P Emery, Habiba Hassouna, Rita Obeid, Mudita Bhugra, Robert A Bonomo, Zeina Kanafani

**Affiliations:** 1 Case Western Reserve University, Cleveland, Ohio; 2 American University of Beirut Medical Center, Beirut, Beyrouth, Lebanon; 3 Spectrum Health, Grand Rapids, Michigan; 4 Spectrum Health/Michigan State University, Grand Rapids, Michigan; 5 Spectrum Health / MSU College of Human Medicine, Grand Rapids, Michigan; 6 Louis Stokes Cleveland VA Medical Center, Cleveland, OH

## Abstract

**Background:**

The ongoing COVID-19 pandemic has thus far resulted in substantial worldwide mortality. As of November 2020, COVID-19 vaccines became available following Emergency Use Authorization (EUA) issued by the FDA. Recent longitudinal studies published as of March 2021 demonstrated that vaccine hesitancy remains high despite improvements compared to 2020. This study sought to explore the perceptions, beliefs, attitudes, and knowledge surrounding COVID-19 and identify determinants uniquely associated with vaccine hesitancy.

**Methods:**

A cross-sectional electronic survey was created based on CDC & IDSA recommendations. The survey was distributed from March 2021 until June 2021 randomly to faculty members, healthcare workers, and students (≥18 years old) across 3 major academic centers (Case Western Reserve University, Spectrum Health, and the American University of Beirut Medical Center [AUBMC]). Data collected included socio-economic characteristics, demographics, knowledge, and attitudes pertaining to COVID-19 and vaccination. A multivariable regression model was utilized to evaluate for independent associations between variables and vaccination willingness/hesitancy as the primary outcome.

**Results:**

In total, 7,197 participants completed the survey with an overall response rate of 94%. Females constituted 75.7% of the study population. Overall, 87.8% of the study cohort indicated willingness to get vaccinated. Factors associated independently with vaccination hesitancy included: younger age, lower attained education, lower knowledge score, physician recommendation against vaccination, not receiving the influenza vaccine annually, and other beliefs and attitudes as reported in table 1.

Table 1. Independent predictors of COVID-19 vaccine hesitancy among study respondents

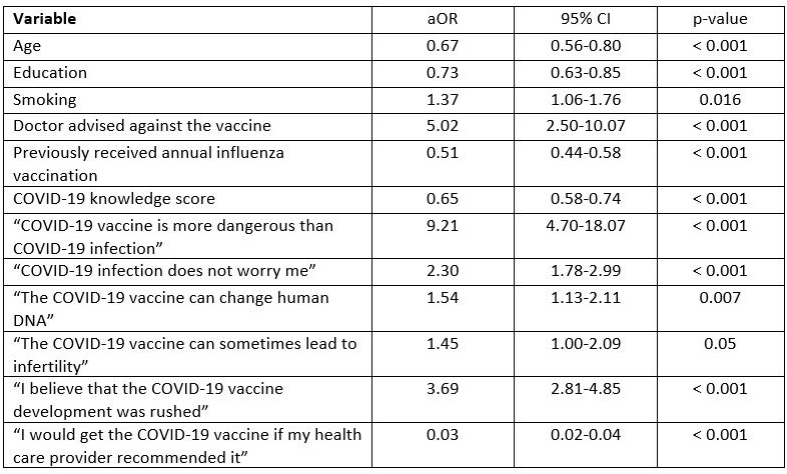

**Conclusion:**

Most survey respondents indicated willingness to receive COVID-19 vaccination. The perception or belief that vaccination is more harmful than COVID-19 disease represented an especially robust barrier against vaccination. Since recommendations made by healthcare providers were strongly associated with either vaccination hesitancy or willingness to get vaccinated, developing educational strategies at this level could enhance vaccine acceptance in an effort to curb the pandemic.

**Disclosures:**

**Robert A. Bonomo, MD**, **entasis** (Research Grant or Support)**Merck** (Grant/Research Support)**NIH** (Grant/Research Support)**VA Merit Award** (Grant/Research Support)**VenatoRx** (Grant/Research Support)

